# Anticancer potential of thiocolchicoside and lauric acid loaded chitosan nanogel against oral cancer cell lines: a comprehensive study

**DOI:** 10.1038/s41598-024-60046-1

**Published:** 2024-04-23

**Authors:** Ameena Mustafa, Meignana Arumugham Indiran, Karthikeyan Ramalingam, Elumalai Perumal, Rajeshkumar Shanmugham, Mohmed Isaqali Karobari

**Affiliations:** 1 Department of Oral Pathology and Microbiology, Saveetha Dental College and Hospitals, Saveetha Institute of Medical and Technical Sciences, Saveetha University, Chennai, Tamilnadu India; 2 Department of Public Health Dentistry, Saveetha Dental College and Hospitals, Saveetha Institute of Medical and Technical Sciences, Saveetha University, Chennai, Tamilnadu India; 3 Center for Global Health Research, Saveetha Medical College and Hospitals, Saveetha Institute of Medical and Technical Sciences, Saveetha University, Chennai, Tamilnadu India; 4 Department of Restorative Dentistry and Endodontics, Faculty of Dentistry, University of Puthisastra, Phnom Penh, 12211 Cambodia

**Keywords:** Thiocolchicoside and lauric acid, Nano formulation, Oral cancer, Inhibition of cell proliferation, Apoptosis, Oral cancer, Nanotechnology in cancer

## Abstract

The present study explored the anticancer activity of a Chitosan-based nanogel incorporating thiocolchicoside and lauric acid (CTL) against oral cancer cell lines (KB-1). Cell viability, AO/EtBr dual staining and Cell cycle analysis were done to evaluate the impact of CTL nanogel on oral cancer cells. Real-time PCR was performed to analyze proapoptotic and antiapoptotic gene expression in CTL-treated KB-1 cells. Further, molecular docking analysis was conducted to explore the interaction of our key ingredient, thiocolchicoside and its binding affinities. The CTL nanogel demonstrated potent anticancer activity by inhibiting oral cancer cell proliferation and inducing cell cycle arrest in cancer cells. Gene expression analysis indicated alterations in Bax and Bcl-2 genes; CTL nanogel treatment increased Bax mRNA expression and inhibited the Bcl-2 mRNA expression, which showed potential mechanisms of the CTL nanogel's anticancer action. It was found that thiocolchicoside can stabilize the protein's function or restore it as a tumour suppressor. The CTL nanogel exhibited excellent cytotoxicity and potent anticancer effects, making it a potential candidate for non-toxic chemotherapy in cancer nanomedicine. Furthermore, the nanogel's ability to modulate proapoptotic gene expression highlights its potential for targeted cancer therapy. This research contributes to the growing interest in Chitosan-based nanogels and their potential applications in cancer treatment.

## Introduction

Head and neck squamous cell carcinoma is the sixth leading cancer worldwide^1^. At the same time, Oral Squamous Cell Carcinoma (OSCC) is the most commonly occurring malignancy among head and neck malignancies, and finding effective treatment and patient management has always been a significant priority^2^. In 2020, Oral cancer was the 11th most common malignancy in men and the 18th most common malignancy in women worldwide. Globally, oral cancer accounted for approximately 377,000 new cancer cases in 2020, with a mortality rate of almost 50%, according to data given by the Global Cancer Observatory (GCO)^3^. Timely detection and proper evaluation are significant as the treatment approaches, outcomes, and patient survival vary significantly^1^. Oral squamous cell carcinoma treatment includes surgery, radiation therapy, chemotherapy, targeted therapy, and immunotherapy. Abundant clinical trials have been conducted to improve treatment efficacy, unmask genetic markers for a complete cure, and increase oral cancer patients' disease-free survival rate^4^. Chemotherapy was the most frequently used (36.8%), while targeted therapy and immunotherapy each constituted 15.2% of the treatment strategies^5^. Patients with advanced or recurrent Oral squamous cell carcinoma (OSCC) are usually treated with surgery, radiotherapy and chemotherapy. Recently, targeted therapies, particularly Ferroptosis, have gained significant attention as potential treatment strategies^6^. Nanoparticles-loaded natural products and their adjuvants can induce redox reactions and immunogenic effects in oral cancer cells and suppress immunosuppressive mechanisms in the tumour microenvironment^2^.

The modification of natural products is a crucial area for the efficient generation of highly potent molecules. Thiocolchicoside, a commonly prescribed muscle relaxant, treats muscle spasms and pain. Derived from colchicine, a natural compound obtained from the plant Gloriosa superba. The chemical composition of thiocolchicoside is represented by the(s)-N-[3-(B-d-glucopyranoxyloxy)-5,6,7,9-tetrahydro-1,2-dimethoxy-10-methylthio)-9-oxobenzo [a]heptalen-7yl] acetamide structure^7–9^. Thiocolchicoside, derived from colchicoside, a naturally occurring glucoside in Colchicum, is classified as a semi-synthetic sulfur derivative. Thiocolchicoside is known for its ability to act as a GABA receptor agonist in the central nervous system, leading to muscle relaxation, analgesic effects, and anti-spasmodic properties. It works by selectively binding to inhibitory gamma-aminobutyric acid and glycinergic receptors and has an agonistic action at spinal-strychnine-sensitive receptors, which mediate its muscle relaxant effect. As a result of these therapeutic qualities, it is prescribed to treat orthopaedic, traumatic, and rheumatologic conditions^7,8,10,11^. Conditions such as fibromyalgia and multiple sclerosis and those associated with muscle spasms and pain are treated with thiocolchicoside. Thiocolchicoside works by blocking the release of certain neurotransmitters in the spinal cord and brain, which helps to reduce muscle spasms and pain. Thiocolchicoside is generally well-tolerated, with few side effects. In addition to its use as a muscle relaxant, thiocolchicoside was found to have other therapeutic properties under research. It showed anticancer effects by downregulating the NF-κB pathway and regulating gene products linked to inflammation and cancer. Thiocolchicoside also suppresses osteoclastogenesis induced by receptor activators of NF-κB ligand (RANKL) and tumour cells. These properties make it a potential candidate for future research in treating cancer and bone-related disorders^11–14^.

Lauric acid is a saturated medium-chain fatty acid found naturally in various plant oils and animal fats, including coconut and palm kernel. It is also present in human breast milk and cow's milk. Lauric acid has many health benefits, including antibacterial, antiviral, antifungal, and anticancer properties. It is extracted from coconut oil to develop monolaurin, an antimicrobial agent that can fight bacteria, viruses, and yeasts. It also has various pharmacological properties under research, such as its role in blood sugar control, blood pressure management and ketone body formation. Despite these studies, few studies reported the anticancer activities of lauric acid against breast and colon cancer cells^15^. Lauric acid reduced Colon cancer cells (Caco2) and IEC-6 cells in the G0/G1 phase, resulting in S and G2/M cell cycle arrest. Lauric acid induces apoptosis in IEC-6 cells by generating high levels of reactive oxygen species and a concomitant decrease in intracellular reduced glutathione levels. Though the exact cellular signalling mechanism underlying lauric acid's anticancer activity is unclear, it may be due to oxidative stress-induced apoptosis^15,16^. Within this field, there is a focus on studying lauric acid-based related compounds to explore their potential as anticancer agents and antioxidants.

Additionally, computational investigations are conducted to understand further and enhance their properties. Lauric oils are characterized by their significant lauric acid content (12:0 or dodecanoic acid) as the predominant fatty acid. Historically, coconut oil has been the primary and most well-known source of lauric oil, obtained from the copra, the inner meat of coconut^17–19^. Verma et al. found the result of anticancer activity of various concentrations of Virgin Coconut Oil (VCO), Fractionated Coconut Oil (FCO) and Processed Coconut Oil (PCO) to be different when treated with oral cancer (KB cell line) cells. 80% PCO showed significant anticancer activity against the KB cell line, which may be attributed to its varied fatty acid composition^20^.

One of the most promising nanotechnology applications is in medicine, and nanoparticles can target particular cells in the body and deliver drugs directly to the disease site^21^. This approach can potentially reduce side effects and improve the efficacy of treatments for cancer and other diseases^22^. Polymeric nanoparticles possess a high level of chemical reactivity and can carry a substantial amount of drugs on their surface due to their small size. However, their small size makes them vulnerable to deposition and oxidation, which can result in losing their desired properties. Stabilizing agents like polymers, surfactants, and polysaccharides safeguard these nanoparticles^23–26^. The effectiveness of these nanoparticles as catalysts and their potential for cytotoxicity are influenced by factors such as the type of polymer used, as well as the size and shape of the nanoparticles. One effective method of stabilization involves encapsulating them within nanogels. The study "Polymeric Nanoparticles as Promising Tool for Anticancer Therapeutics" delves into the application of polymeric nanoparticles in cancer therapies, offering valuable insights into their potential role in combating cancer^16–18^.

Chitosan is a chitin-derived biopolymer found in crustacean shells, as in shrimp and crabs. As a natural, non-toxic, and biodegradable substance, chitosan has various applications in various industries, including agriculture, food, and Pharmaceuticals. It inhibits bacterial and fungal growth effectively and reduces fat content in food products. In the pharmaceutical industry, chitosan is used as a drug delivery system and is efficacious in improving drug bioavailability and stability and reducing toxicity. It is also used in wound healing and tissue engineering, as it has been found to have antimicrobial and anti-inflammatory properties, as well as the ability to promote tissue regeneration^27,28^.

Nanogels comprise a wide range of naturally occurring or synthetic polymers or both. The polymer networks that form nanogels contain various functional groups, including hydroxyl (-OH), amine (-NH2), carboxyl (-COOH), and carbonyl (–C=O) groups^29^. These functional groups play a significant role in the unique properties of nanogels, such as their stimuli-responsiveness and drug-loading capacity^30,31^. Overall, the composition of nanogels and the functional groups in their polymer networks greatly influence their properties and potential applications.

The synthesis of nanogels is an active area of research due to their potential for use as drug-delivery systems^32^. Nanogels are composed of polymer networks that contain various functional groups, which play a significant role in their unique properties.The Fourier transform infrared (FTIR) method is a valuable tool for characterizing nanogels, as it can provide detailed information about their chemical composition and structure by the rotation and vibration of molecules affected by infrared radiation at a specific wavelength. The characteristic peaks observed in the spectrum confirmed the success of the synthesis of the nanogel and its chemical composition and structure. The peaks corresponded to various functional groups, such as hydroxyl (–OH), amine (–NH2), carboxyl (–COOH), and carbonyl (–C=O) groups, that are present in the polymer networks of nanogels^33^. Thiocolchicoside and lauric acid have significant pharmacological potential, and the nanogel formulation was expected to have excellent therapeutic effects against oral cancer. In the present study, we explored the anticancer activity of Thiocolchicoside-lauric acid nanogel synthesized using chitosan as a stabilizing agent in oral cancer cell lines.

## Material and methods

### Nanogel preparation protocol

CTL nanogel was prepared according to the protocol given by Ameena et al.^32^. The lauric acid solution was prepared by dissolving 0.5 g of lauric acid in 10 mL of ethyl alcohol, While the thiocolchicoside solution was prepared by dissolving 50 mg of thiocolchicoside in 10 mL of distilled water. Three more different formulations of Lauric acid and Thiocochicoside were prepared: 1gm Lauric acid and 50 mg Thiocolchicoside, 0.5gm Lauric acid and 100 mg Thiocolchicoside, and 0.25 g Lauric acid and 150 mg Thiocolchicoside respectively.

The solutions were uniformly mixed using a vortex mixer for 15 min and kept in an orbital shaker overnight at 110 rpm. Chitosan was prepared by adding 0.5 g of medium molecular weight chitosan to 49.5 mL of distilled water. 0.5 mL of glacial acetic acid was added, and the resultant chitosan mixture was stirred continuously for about 2–3 h using a magnetic stirrer at 800 rpm to form a clear solution. The Thiocolchicoside-Lauric acid nanogel was prepared by adding 5 mL of prepared lauric acid solution to 5 mL of prepared thiocolchicoside solution. The reaction mixture was kept on a magnetic stirrer at 700 rpm for 2 h. 10 mL of medium molecular-weight chitosan was added, and the chitosan-mediated nanogel was stirred for up to 24 h to attain uniform dispersion.

### In-vitro drug release study

The in vitro release study of CTL nanogel from the matrix was determined using a modified dissolution basket-type apparatus in a two-sided open glass cylinder. The dialysis membrane (Hi Media Mol. Wt. 12-14 k) was fixed on one end, and the cylinder was filled with 1 ml nanogel on the other end. The saliva and Plasma with pH ranging from 6.8 to 7.4 were used as a dissolution medium and filled in a mixing bowl of around 200 ml. The temperature was maintained at 37 ± 1 °C by circulating hot water through the jacket. The 0.5 mL samples were withdrawn at scheduled time intervals (0.5, 1, 2, 3, 4, 5, 6, 7, 8, 9, 10, 11, 12 h) and were replaced with the same volume of Saliva and Plasma to maintain the sink condition. Samples were analysed using a UV–visible spectrophotometer.

### Cell line maintenance

Oral cancer cell lines (KB-1) were obtained from the NCCS, Pune. The cells were grown in T25 culture flasks containing Dulbecco's Modified Eagle Medium supplemented with 10% FBS and 1% antibiotics. Cells were then maintained at 37◦C in a humidified atmosphere containing 5% CO2. Upon reaching confluency, the cells were trypsinized and passaged.

### Cell viability (MTT) assay

The cell viability of 4 different formulations of CTL nanogel treated with oral cancer (KB-1) cells was assessed by MTT assay. The assay is based on the reducing capacity of metabolically active cells to convert soluble yellow tetrazolium salt to insoluble purple formazan crystals. KB-1 cells were plated in 96 well plates at 5 × 10^3^ cells/well concentrations. The cells were washed twice with 100 μl of serum-free medium 24 h after plating, and the cells were made to starve by incubating them in serum-free medium at 37ºC for 3 h. Then, cells were treated with different concentrations of CTL nanogel for 24 and 48 h. At the end of treatment, the medium from control and CTL nanogel-treated cells were discarded, and 100 μl of MTT containing DMEM (0.5 mg/ml) was added to each well. The cells were then incubated at 37ºC in the CO_2_ incubator for four hours. The MTT medium was discarded, and the cells were washed with 1 × PBS. Then, the formazan crystals formed were dissolved in dimethyl sulfoxide (100 μl) and incubated in the dark for one hour. The developed colour intensity was assayed using a Micro ELISA plate reader at 570 nm. The viable cell count was expressed as a percentage of control cells cultured in a serum-free medium. Cell viability in the control without any treatment was considered 100%. The cell viability was calculated using the formula: Percentage cell viability = [A570 nm of treated cells/A570 nm of control cells] × 100.

### Morphology study

Based on the MTT assay, we selected the optimal doses, 5 and 10 µl/ml (IC-50:10.29 µl/ml) for further studies. A Phase contrast microscope analyzed morphologic changes of cells. 2 × 10^5^ cells were seeded in 6 well plates and treated with five and 10 µl/ml of CTL nanogel for 24 h. The medium was removed at the end of the incubation period, and cells were washed once with a phosphate buffer saline (PBS) at pH 7.4. The plates were then observed under a phase contrast microscope.

### Cell cycle analysis by flow cytometry

The KB-1 cells (1 × 10^6^ cells/per plate) were cultured in 100-mm culture plates containing growth medium. After starvation, the cells were treated with Chitosan thiocolchicoside lauric acid Nanogel (5 and 10 µl/ml) for 24 h, and then the cells were harvested with 0.25% trypsin and centrifuged at 3000xg for 5 min. Then, the cells were washed with PBS. After centrifugation, the cells were fixed in 70% ice-cold ethanol overnight at − 20 °C. The cells were then incubated in 50 μg/ml of propidium iodide in PBS and 1 mg/ml of ribonuclease in PBS for 30 min. Cell cycle analyses were performed on a BD FACSCanto^TM^II (Becton and Dickinson Biosciences, Mountain View, CA, USA), and the data were analyzed using BD FACSCanto clinical software.

### ROS expression level using DCFH-DA staining.

The intracellular ROS level in treated KB-1 cells was analyzed by DCFDA staining. The cells were grown in 24 well plates and treated with CTL gel (5 and 10 µl/ml) for 24 h time points. After the nanogel treatment, the cells were incubated with 200 µl of DCFDA (10 µM) working solution at 37 °C for 20 min. After incubation, the DCFH-DA working solution was removed, and cells were washed with PBS and the intracellular ROS level under the fluorescence microscope.

### Determination of mode of cell death by acridine orange (AO)/ethidium bromide (EtBr) dual staining

The effects of CTL nanogel in KB-1 (5 and 10 µl/ml) cell death were also determined by AO/EtBr dual staining as described previously^34^. The cells were treated with CTL nanogel (10 µl/ml) for 24 h, harvested, and washed with ice-cold PBS. The pellets were resuspended in 5 µl of acridine orange (1 mg/mL) and 5 µl of EtBr (1 mg/mL). The stained cells' apoptotic changes were then observed using a fluorescence microscope.

### Apoptosis assay using Annexin-V flow cytometry analysis.

The KB-1 cells were seeded in a 60-mm dish. IC-50 concentration (10 mg/ml) of CTL nanogel was added, and the cells were cultured for 24 h. Apoptosis of cells was assessed with an Annexin V/FITC Apoptosis Detection kit (BD Biosciences, Franklin Lakes, NJ, USA). The CTL nanogel-treated KB-1 cells were harvested and stained with propidium iodide (PI) and annexin V-FITC and were measured using a FACS Calibur flow cytometer (BD Biosciences).

### Real-time PCR

The gene expression of proapoptotic/antiapoptotic proteins was analyzed using real-time PCR. The total RNA was isolated by standardized protocol using Trizol Reagent (Sigma). 2 μg of RNA is used for cDNA synthesis using reverse transcription using a PrimeScript, 1st strand cDNA synthesis kit (Takara, Japan). The targeted genes were amplified using specific primers. The primer sequences BAD—Forward: 5′gctggacattggacttcctc3′ Reverse: 5′ctcagcccatcttcttccag3′. BCL-2-Forward: 5′gctggacattggacttcctc3′ Reverse: 5′ctcagcccatcttcttccag3′. GAPDH-Forward: 5′cgaccactttgtcaagctca 3′ Reverse: 5′ cccctcttcaaggggtctac 3′. The PCR reaction was performed with iTaq, Universal SYBR green supermix (Bio-Rad, USA), which contains SYBR green dye and all the PCR components. Real-time PCR was performed in an MX3000p (Stratagene, Europe). The results were analyzed using the comparative C_T_ method, and the 2^−∆∆CT^ method was used for the fold change calculation described by Schmittgen and Livak^35^.

### Molecular docking studies of thiocolchicoside against therapeutic cancer targets

The X-ray crystal structures of the target proteins, namely PAK4 (PDB ID—5XVA), TP53 wildtype (PDB ID—3KMD), and TP53 Y220C mutant (PDB ID—5O1A), were obtained from the Protein Data Bank (PDB). Using the mutagenesis tool in PyMOL, we introduced two hotspot mutations, R175H (a mutation affecting DNA contact) and R248Q (a mutation affecting protein structure), into the TP53 wildtype structure. These mutated structures were then saved in PDB format. Subsequently, all five protein targets (PAK4, TP53 wildtype, TP53 Y220C, TP53 R175H, and TP53 R248Q) underwent preprocessing using both PyMOL and the Swiss PDB Viewer. In AutoDock, the Macromolecules were imported, Hydrogen atoms were appended to the polar residues, Atom types were assigned to AD4 classification, Kollman charges were applied to the atoms, and the net charge was distributed accordingly. The ligand thiocolchicoside and its structure were obtained from PubChem in SDF format and converted into PDBQT format. This ligand was then incorporated into AutoDock, where the torsion roots were identified.

The grid box was created based on the amino acid residues interacting with the co-crystal ligand in PAK4 (PDB ID—5XVA) and TP53 Y220C mutant (PDB ID—5O1A) structures. Ligand binding sites for TP53 wildtype (PDB ID—3KMD), TP53 R175 mutant, and TP53 R248Q mutant were predicted using the Prankweb tool. The top-ranked binding sites from these predictions were used to generate the grid for molecular docking. The molecular docking simulations were conducted using the AutoDock algorithm, which involved running 20 genetic search algorithm iterations for each protein target. Subsequently, the docked complex with the highest binding energy was retrieved and subjected to interaction analysis. The docking procedure's reliability was confirmed by re-docking the co-crystal ligands from PAK4 (PDB ID—5XVA) and TP53Y220 (PDB ID—5O1A). The resulting root-mean-square deviation (RMSD) values were 0 and 0.056, respectively.

### Statistical analysis

All data obtained were analyzed by One way ANOVA flowed by Students-t-test using SPSS, represented as mean ± SD for triplicates. The level of statistical significance was set at *p* < 0.05.

## Results

### Characterisation of Chitosan thiocolchicoside-lauric acid nanogel

#### UV spectroscopy

**Figure 1:**
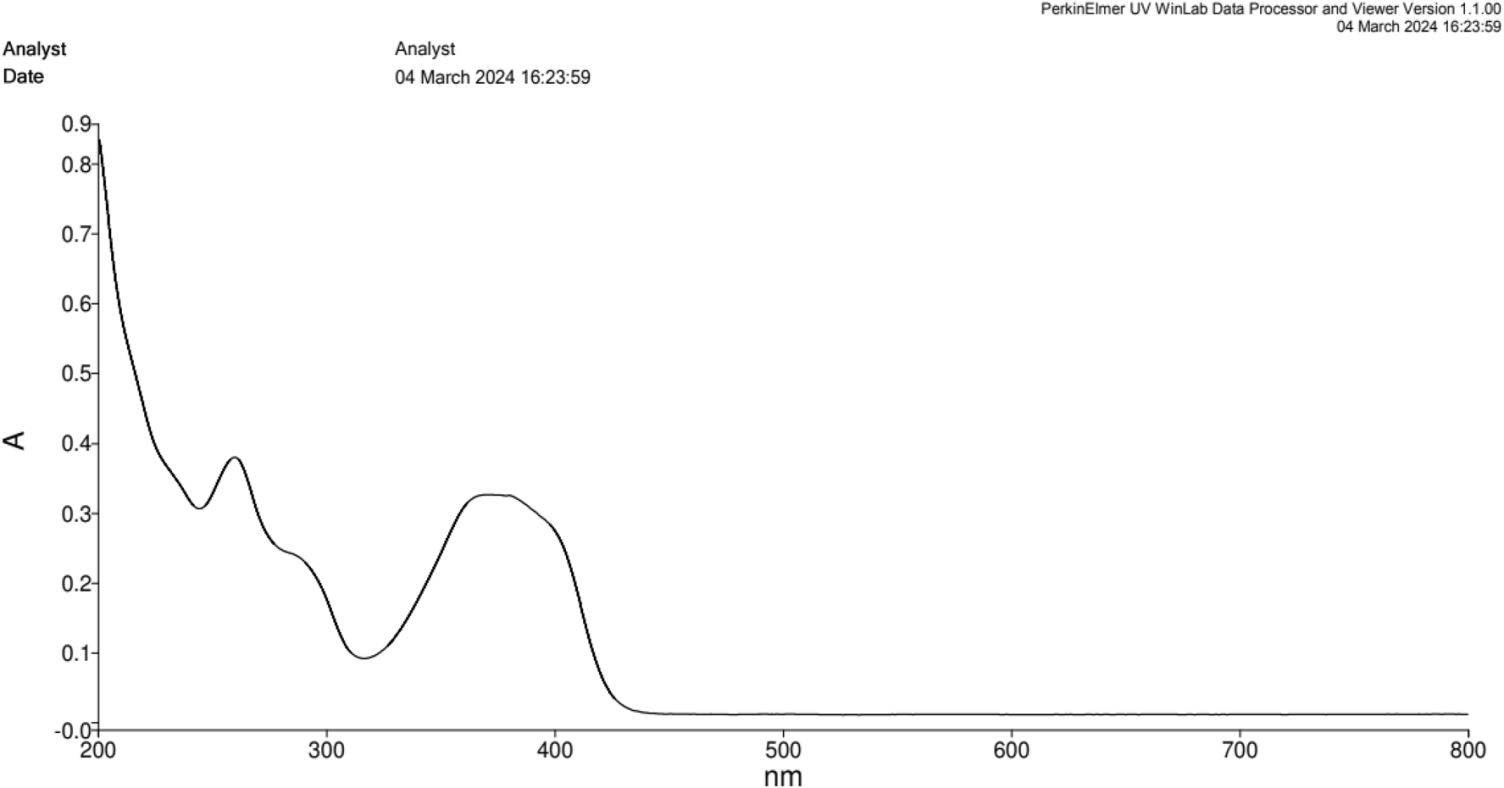
UV spectroscopy—CTL nanogel.

The preliminary and quantitative analysis confirmed using a UV visible absorption spectrum, the obtained multiple absorption peaks of chitosan thiocolchicoside-lauric acid nanogel as shown in Fig. 1. The absorption peaks at ~ 250 nm corresponding to C–C bonds (in the presence of chitosan (C–C) bonds), 291 nm representing sp3 hybridization and 380 nm corresponding to aromatic sulphur interaction between the intermolecular interaction, and charge transfer between thiocolchicoside- to chitosan bonds.

#### Fourier Transform Infrared Spectroscopy (FT-IR)

**Figure 2:**
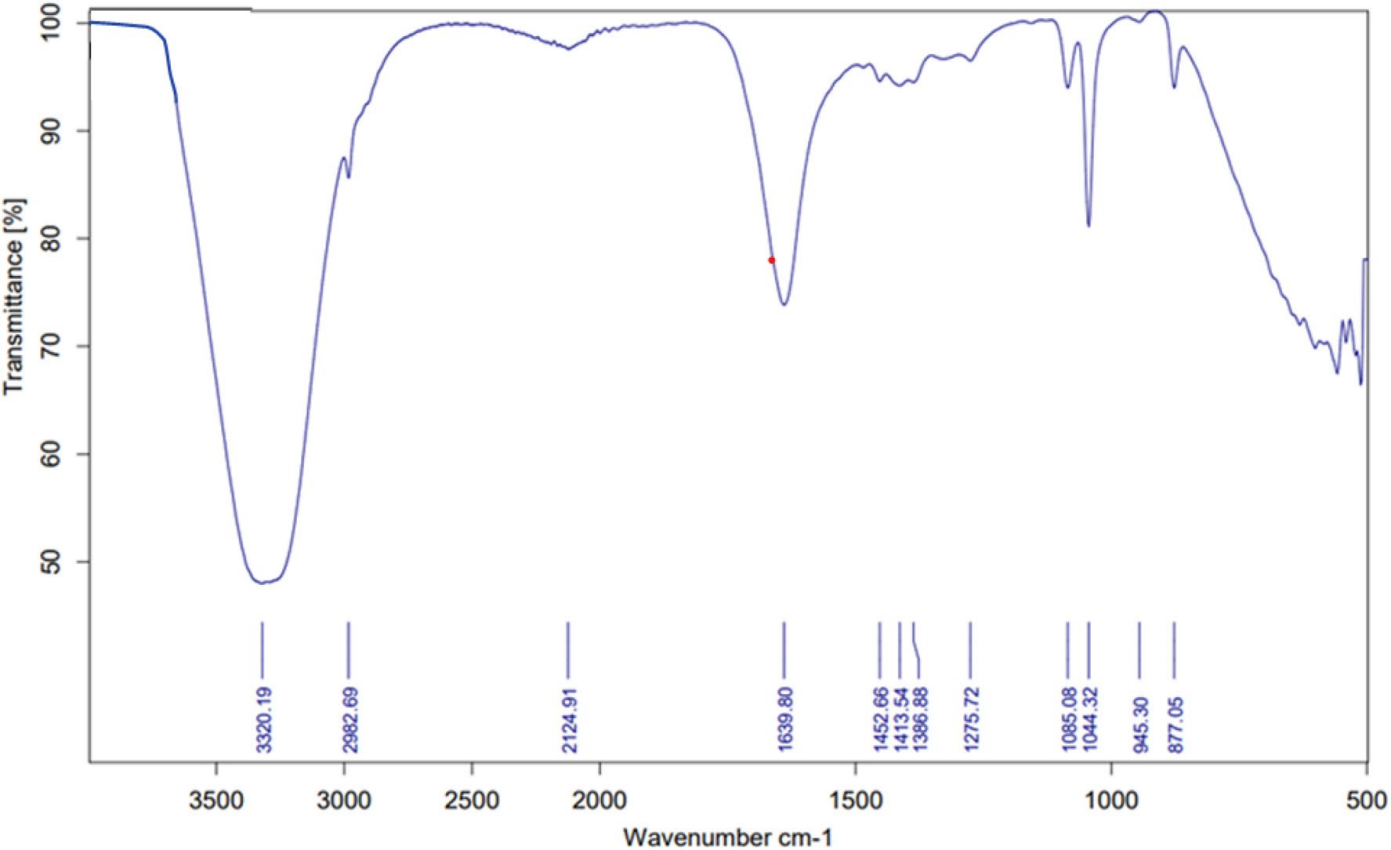
FT-IR spectra of CTL nanogel.

**Figure 3:**
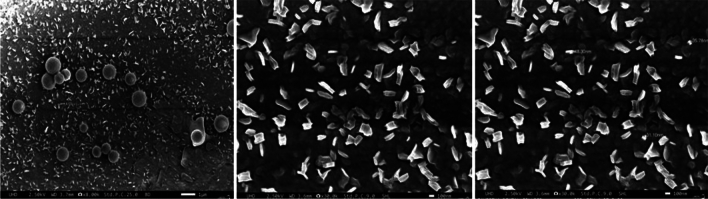
Scanning microscopic image of CTL nanogel showing the size of nanoparticles.

The CTL nanogel was characterized using Fourier Transform Infrared Spectroscopy (FT-IR). The FT-IR spectrum of CTL nanogel showed characteristic peaks at 3320.19, 2982.69, 2124.91, 1639.80, 1452.66, 1413.54, 1386.88, 1275.72, 1085.08, 1044.32, 945.30, and 877.05 cm^−1^. The peak at 3320.19 cm^−1^ was assigned to the stretching vibration of the hydroxyl (–OH) group, while the peaks at 2982.69 and 2124.91 cm^−1^ were given to the stretching vibrations of the aliphatic –CH3 and –CH2 groups, respectively. The peak at 1639.80 cm^−1^ corresponded to the stretching vibration of the carbonyl (C=O) group in the amide bond. The 1452.66, 1413.54, and 1386.88 cm^−1^ peaks corresponded to the bending vibrations of the –CH2 and –CH3 groups. The peak at 1275.72 cm^−1^ is assigned to the stretching vibration of the ester (–COO–) group, and the peak at 1085.08 cm^−1^ corresponded to the stretching vibration of the ether (C–O–C) group. Finally, the peaks at 1044.32, 945.30, and 877.05 cm^-1^ corresponded to the stretching vibrations of the C–O, C–C, and C–H bonds, respectively (Fig. 2). These characteristic peaks confirmed the successful synthesis of the CTL nanogel and provided insights into the chemical composition and structure of the nanogel (Fig. 3).

#### Dynamic light scattering (DLS)

**Figure 4:**
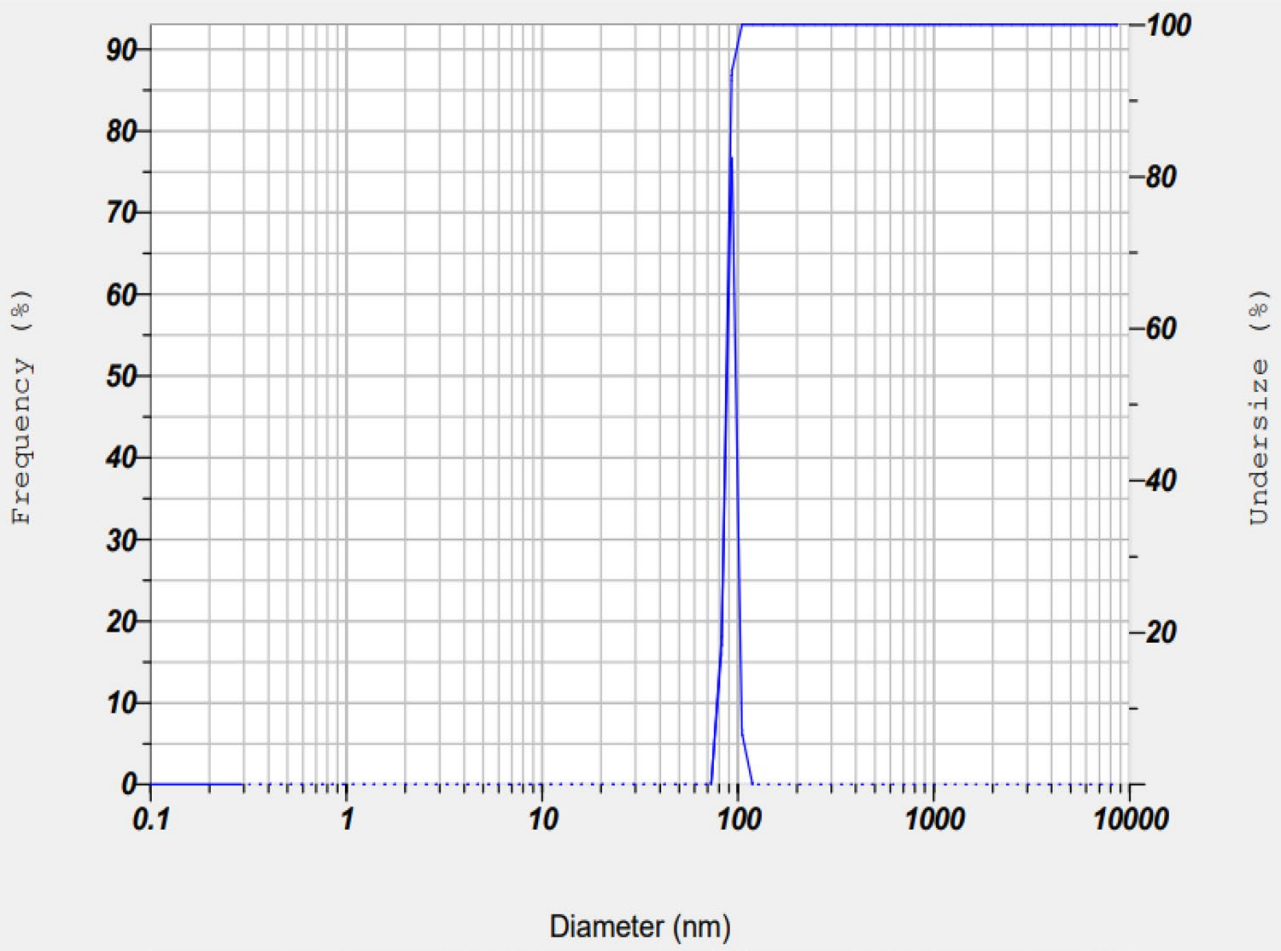
Dynamic light scattering of CTL nanogel.

While scattering the light intensity in the monodispersed CTL nanogel, verified the average particle size in the 70–110 nm range under the following temperature of 24.9 °C and Viscosity of 0.897 mPa s, as illustrated in Fig. 4, dynamic light scattering investigations optimized the average size of the as-prepared CTL nanogel. The degree of the detecting angle was 90 degrees. The degree angle verifies the PMT detector's accuracy in determining the particle size range of monodispersed CTL nanogel layer by layer.

#### Zeta potential

**Figure 5:**
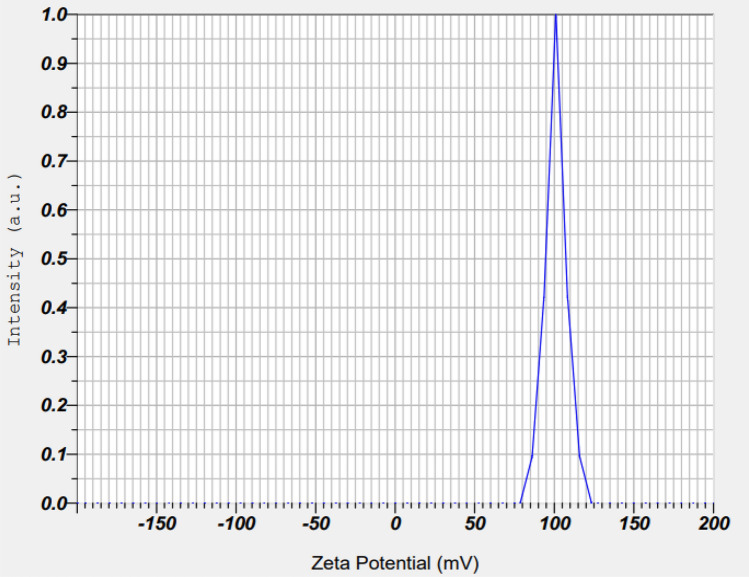
Zeta potential of CTL nanogel.

The zeta potential of a CTL nanogel refers to its surface charge, which is crucial for its stability and interaction with other molecules in solution. A higher absolute zeta potential value typically indicates greater electrostatic repulsion between nanoparticles, leading to increased stability. Based on this, our results confirmed the outermost active surface charge of ~ 101 mV, as shown in Fig. 5. The zeta potential value of more than ± 30 mV confirms the highly stable active surface of the outermost layer in the CTL nanogel formation.

#### Drug loading and release studies

**Figure 6:**
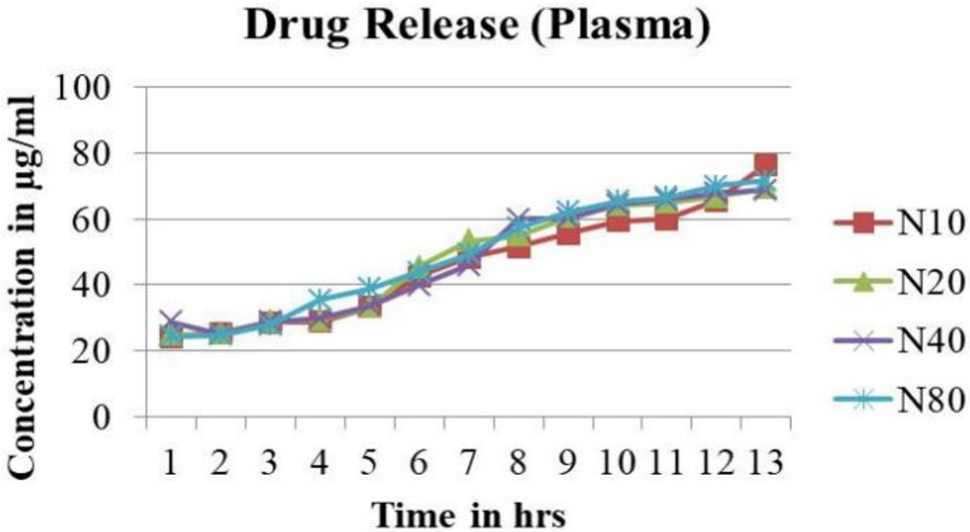
CTL nanogel release in Plasma.

**Figure 7:**
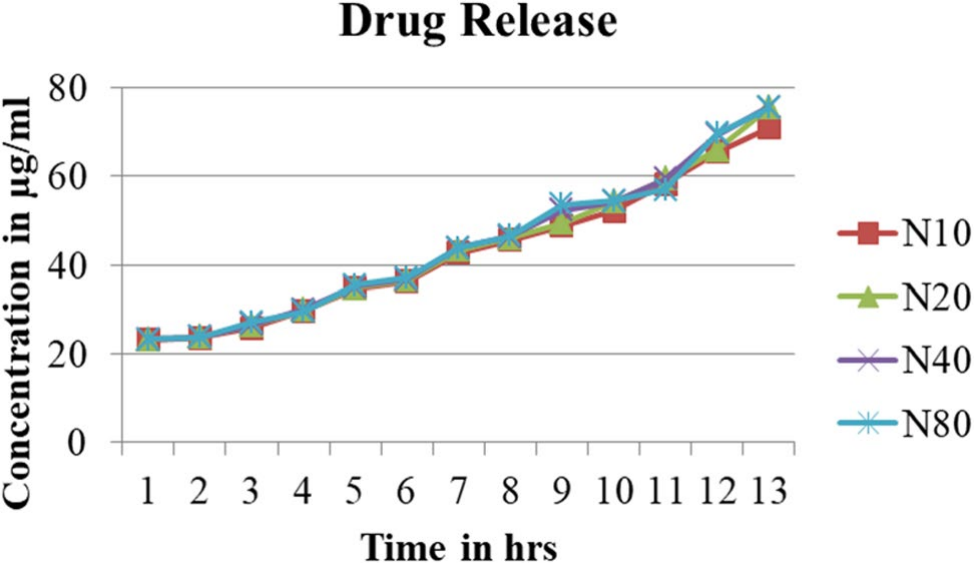
CTL nanogel release in Saliva.

In-vitro release of CTL nanogel from the matrix at different concentrations (10, 20, 40, 80 µg/ml) are shown in Figs. 6 and 7. There was a decrease in the percentage of release from nanogel with a concomitant increase in strength due to the firm matrix structure created by nanogel. The lower the polymer concentration, the less viscous the matrix was and the lower the matrix strength; thus, the faster the drug release from the matrix with a shorter duration of drug release, as shown in Tables 1 and 2.

**Table 1:** *In-vitro* release profile of CTL Nanogel in Plasma.

Time (h)	% Cumulative release in plasma			
	CTL nanogel (10 µg/ml)	CTL nanogel (20 µg/ml)	CTL nanogel (40 µg/ml)	CTL nanogel (80 µg/ml)
0.5	24.16	24.82	28.82	24.32
1	25.38	25.43	24.99	24.82
2	28.71	28.77	28.88	27.77
3	28.88	29.43	29.93	35.49
4	33.27	33.27	33.71	38.88
5	42.71	45.49	39.82	43.82
6	48.22	53.27	45.93	49.38
7	51.60	54.93	59.88	57.38
8	55.54	60.60	60.21	62.21
9	59.32	64.21	64.38	65.43
10	60.04	64.93	66.04	66.66
11	65.54	66.77	67.82	69.93
12	76.49	69.38	68.77	71.60

**Table 2:** In vitro release profile of CTL Nanogel in Saliva.

Time (h)	% cumulative release in Saliva			
	CTL nanogel (10 µg/ml)	CTL nanogel (20 µg/ml)	CTL nanogel (40 µg/ml)	CTL nanogel (80 µg/ml)
0.5	23.04	23.16	23.21	23.21
1	23.54	23.71	23.71	23.71
2	25.82	26.49	26.66	27.10
3	29.71	29.88	29.99	29.43
4	34.66	34.82	35.04	35.43
5	36.27	36.54	36.66	37.10
6	42.71	43.32	43.77	43.82
7	45.49	45.99	46.38	46.54
8	48.82	49.38	52.38	53.66
9	52.10	54.21	54.21	54.43
10	58.21	59.32	59.32	57.04
11	65.43	66.04	69.38	69.60
12	70.99	75.49	75.54	75.49

#### Entrapment efficiency

**Figure 8:**
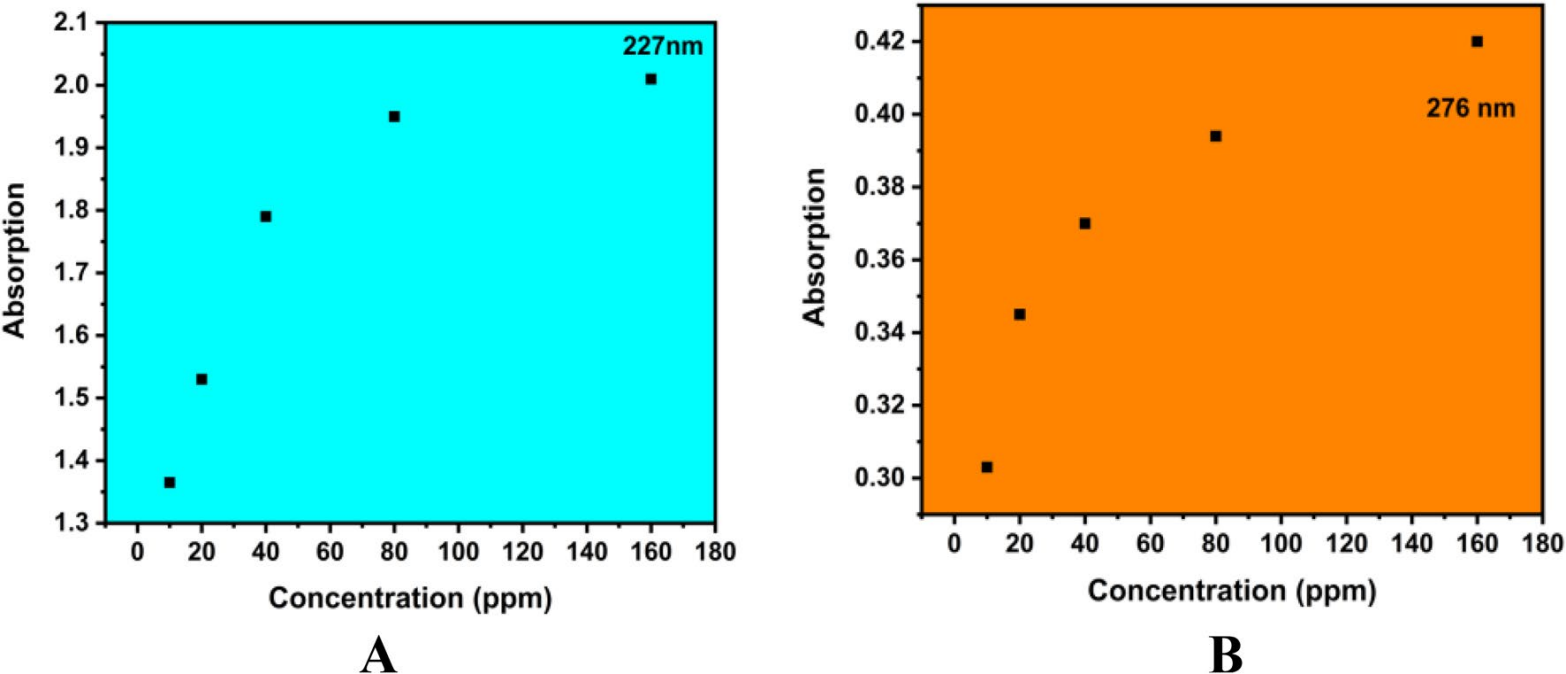
Entrapment efficiency of CTL nanogel (**A**) 227 nm, (**B**) 276 nm.

UV visible absorption spectra (200–400 nm) were recorded in SHIMADZU UV–Visible absorption spectroscopy; the obtained results are shown in Fig. 8. The absorption-dependent Entrapment efficiency confirmed by different concentrations of CTL nanogel is shown in Fig. 8A (227 nm) and Fig. 8B (276 nm). Herein, we observed that the higher concentration played a major role; the sensitivity slope observed at 227 and 276 nm was 0.80509, indicating a linear increasing trend for the Entrapment efficiency of CTL nanogel.

### Effect of CTL nanogel on Cell viability and Cell Morphological Changes of Oral Cancer Cell Line

**Figure 9:**
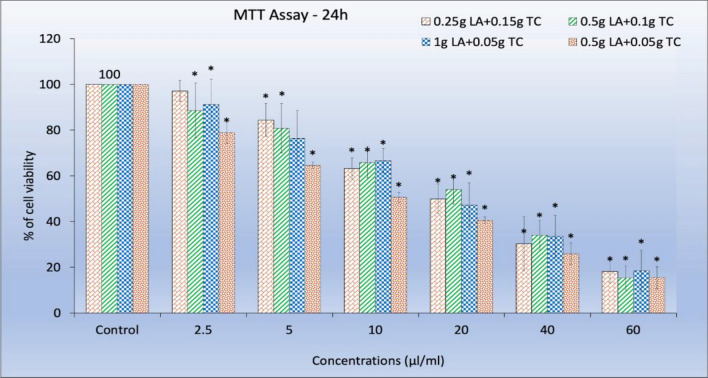
Cytotoxic effects of CTL nanogel on oral cancer cells (KB-1). Cells were treated with CTL nanogel (2.5–60 µl/ml) for 24 h, and cell viability was evaluated by MTT assay. Data are shown as means ± SD (*n* = 3). *Compared with the control group, *p* < 0.05. Control CTL nanogel 5 μl/ml CTL nanogel 10 μl/ml.

**Figure 10:**
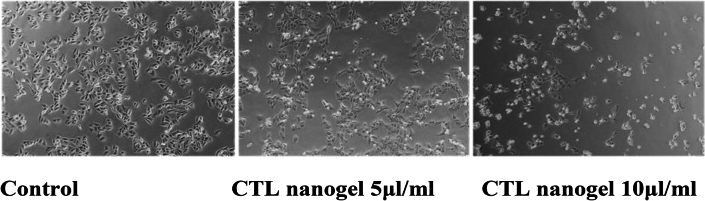
Effect of CTL nanogel on cell morphology of human oral cancer cell line (KB-1). Cells were treated with CTL nanogel (5 and 10 5 μl/ml) for 24 h, and cells were observed under an inverted phase contrast microscope. Human oral cancer cells and the control group were treated with CTL nanogel at 24 h. Images were obtained using an Inverted phase contrast microscope.

The MTT assay assessed the anticancer activity of the four different formulations of CTL Nanogel against oral cancer cells. The nanogel exhibited a significant dose-dependent reduction in cell viability compared to the control group (*p* < 0.05) when treated with four formulations, but the nanogel formulation with 0.5 gm lauric acid and 0.05 gm thiocolchicoside showed the maximum cytotoxicity. Figures 9 and 10 display the morphological changes observed in oral cancer cells (KB-1) after treatment with various nanogel concentrations. Phase-contrast microscopy revealed noticeable alterations, including apoptotic bodies, cell shrinkage, and detachment of cells. The nanogel treatments administered 24 h in oral cancer cells (KB-1) resulted in substantial and dose-dependent cytotoxicity (with a p-value less than 0.001).

### CTL nanogel induces cell cycle arrest in oral cancer cells

**Figure 11:**
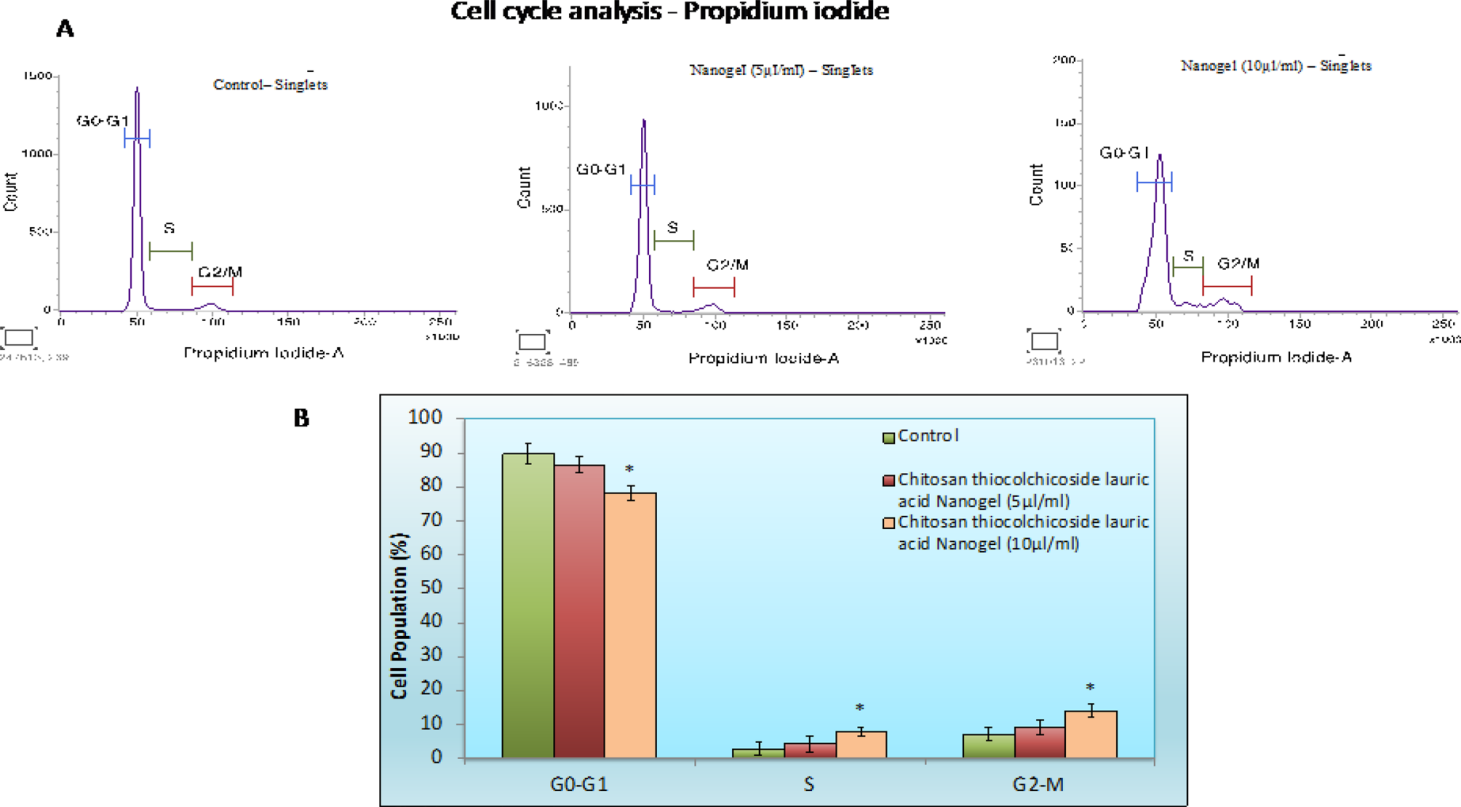
Cell Cycle Analysis: (**A**) flow cytometric analysis of PI-stained oral cancer cells treated with the nanogel revealed alterations in the cell cycle. (**B**) Representative plots showing PI staining of oral cancer cells treated with the CTL Nanogel. Data represent the mean ± SD. of three independent experiments.

Flow cytometric analysis of propidium iodide (PI)–stained oral cancer cells treated with the nanogel revealed alterations in the cell cycle. The nanogel caused cell cycle arrest, with a significant increase in the percentage of cells in the G2/M phase compared to control in response to CTL nanogel treatment (*p* < 0.05). This indicates that the nanogel interferes with cell proliferation, contributing to its anticancer effects (Fig. 11).

### CTL nanogel induces apoptosis in oral cancer cells

**Figure 12:**
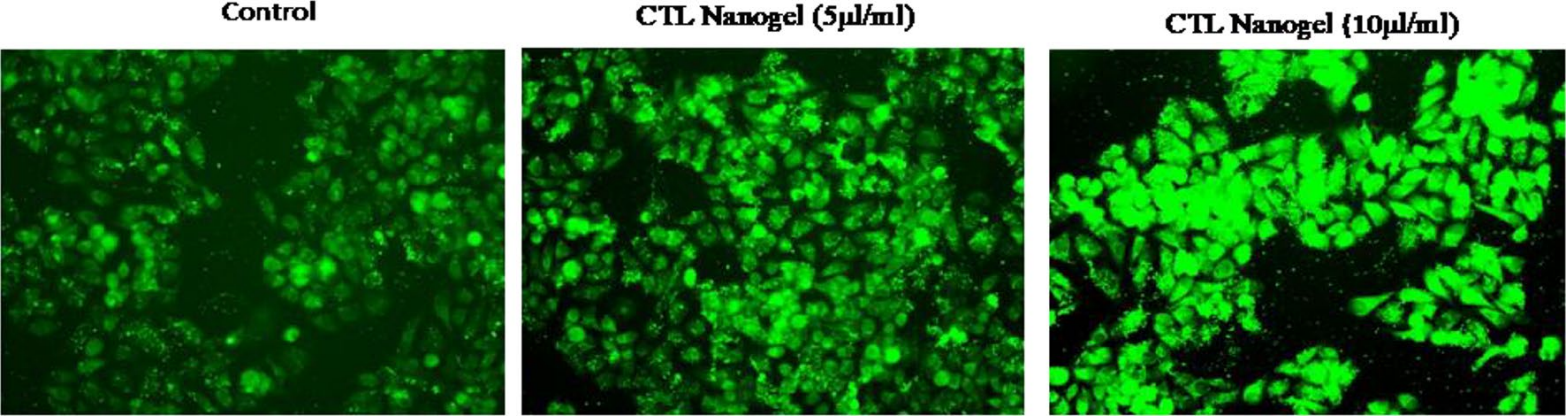
DCFH -DA Staining-CTL Nanogel Effect on oral cancer cells' ROS level. Cells were treated with CTL nanogel for 24 h. Images were obtained using an Inverted fluorescence microscope.

In this study, we investigated the cytotoxic effects on cancer cells and identified an excessive expression of ROS as a potential cause for the observed cytotoxicity. To explore this further, they exposed oral cancer cells (KB-1) to two concentrations of nanogel, specifically 5 and 10 μl/mL, for 24 h. The level of ROS expression was evaluated using DCFH-DA staining, and the results indicated that both nanogel concentrations led to a significant increase in ROS expression in the oral cancer cells (KB-1). Notably, the cells treated with 10 μl/ml exhibited a higher intensity of green fluorescence than those treated with 5 μl/mL of nanogel, as shown in Fig. 12.

**Figure 13:**
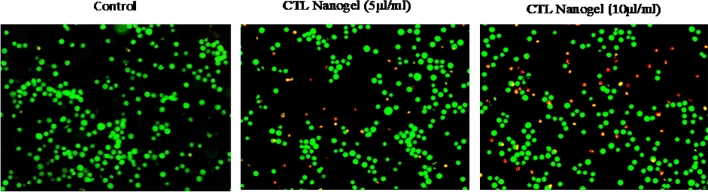
Induction of apoptosis in CTL nanogel-treated cells analyzed by AO/EtBr dual staining in oral cancer cells. Cells and the Control group were treated with CTL nanogel after 24 h. Images were obtained using an inverted fluorescence microscope. The induction apoptosis in CTL Nanogel-treated oral cancer cells was analyzed by annexin V-5-fluorescein isothiocyanate (FITC)/propidium iodide (PI) staining.

Our present investigation utilized the AO/EtBr staining method to evaluate apoptosis-mediated cell death in nanogel-treated cells. This approach allowed us to assess cell nuclei morphology in untreated control cells and those exposed to nanogel. During the staining process, live cells displayed a consistent green colour, early apoptotic cells exhibited a yellow stain, and late apoptotic cells presented condensed and often fragmented nuclei with an incorporated ethidium bromide stain appearing red. The AO/EtBr staining method corroborated our quantitative apoptosis analysis results, providing further evidence that the highest rate of cell death, characterized by nuclear deformation and loss of cell wall integrity, was explicitly observed in KB-1 cancer cells treated with nanogel (Fig. 13).

**Figure 14:**
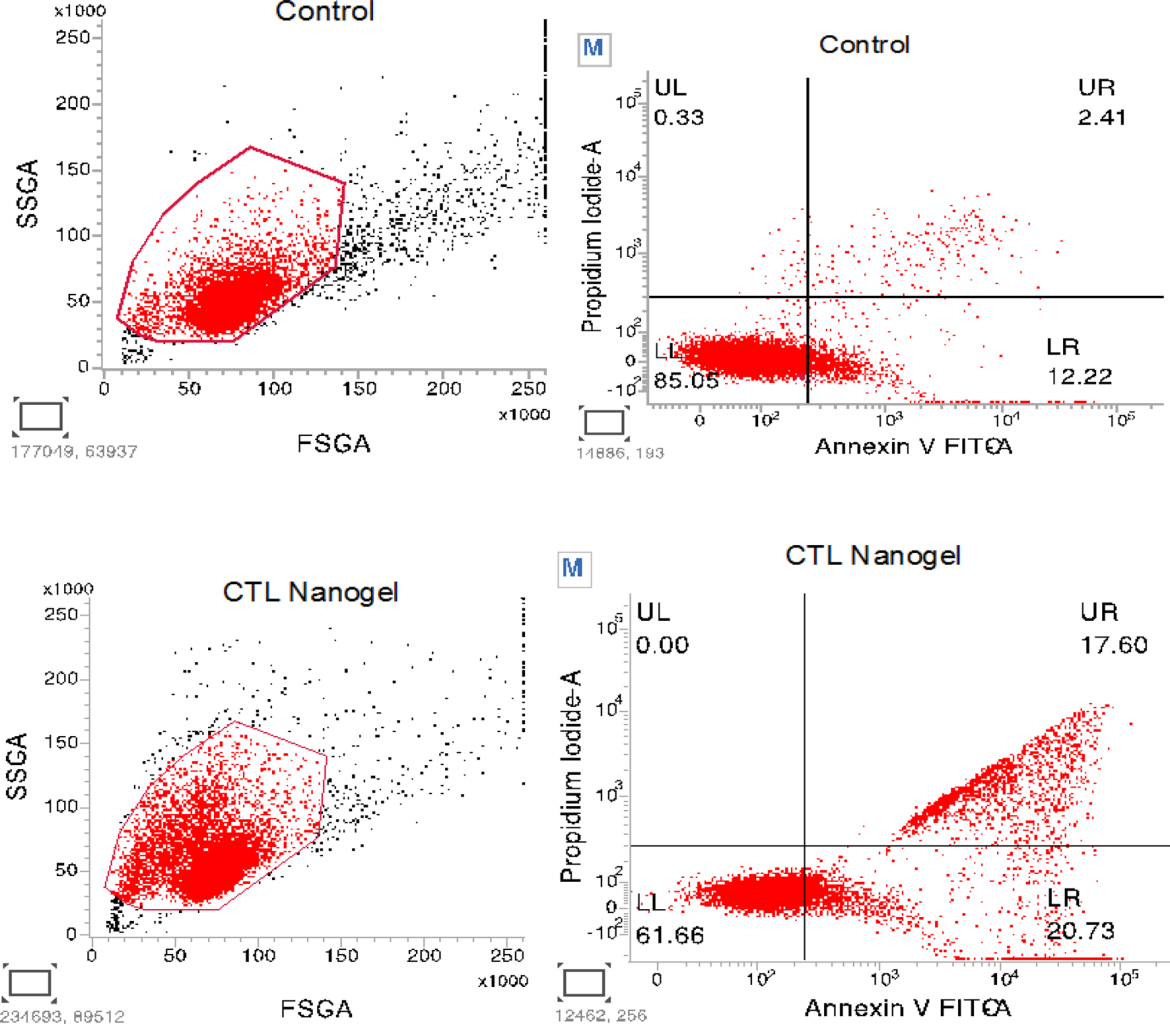
Effect of CTL Nanogel on apoptosis of oral cancer cells. Apoptosis was evaluated using annexin V-5-fluorescein isothiocyanate (FITC)/propidium iodide (PI) staining followed by flow cytometric analysis. (**A**) Representative plots showing annexin V-FITC/PI staining of oral cancer cells treated with the CTL Nanogel (10 μl/ml).

To determine the occurrence of apoptosis in cultured oral cancer cells, we examined both untreated and CTL Nanogel-treated KB-1 cells using Annexin-V and PI. Compared to the untreated group, CTL Nanogel-treated KB-1 cells displayed a higher percentage of apoptotic cells. Specifically, the percentage of apoptotic cells significantly increased in CTL Nanogel-treated KB-1 cells (38.33%, n = 3, P ≤ 0.05, Fig. 11) compared to untreated cells (14.63%). No necrotic signs were observed in CTL Nanogel-treated cell lines, while control cells exhibited only 0.33% necrosis (Fig. 14).

### Upregulation of proapoptotic genes and downregulation of Bcl-2 Gene Expression

**Figure 15:**
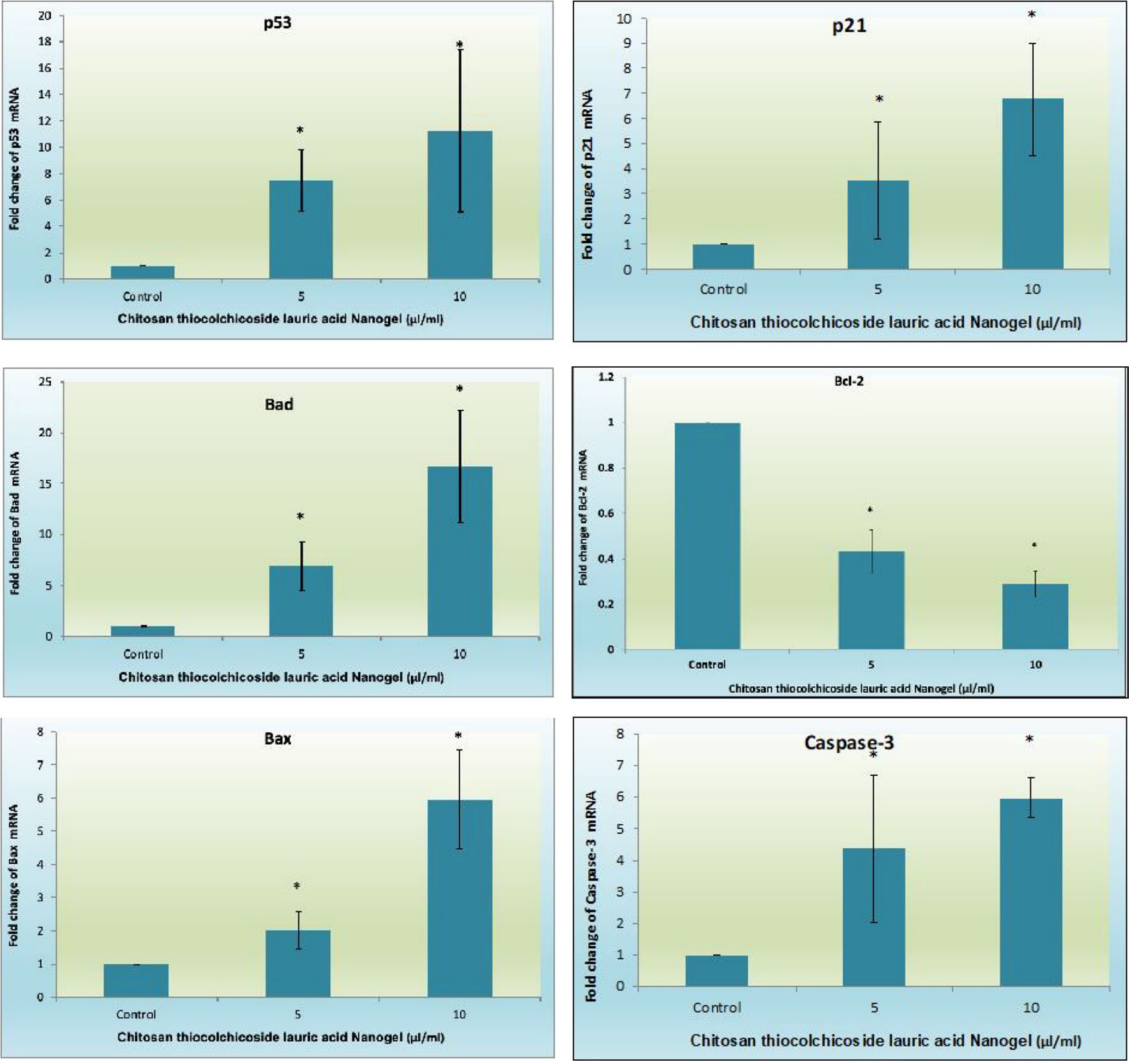
Effects of CTL nanogel on apoptosis signaling molecules gene expression in oral cancer cells. All the gene expression is normalized to GAPDH mRNA expression, and the results are shown as a fold change from the control. Each bar represents the mean + SEM of three independent observations. ‘*’ denotes statistical significance between control versus drug treatment groups at *p* < 0.05.

The expression levels of key genes involved in the intrinsic mitochondrial apoptosis pathway were examined by real-time PCR. After 24 h of nanogel treatment, oral cancer cells showed an upregulation of proapoptotic genes, including Bax, Bad, and caspase 3 (*p* < 0.05). In contrast, the antiapoptotic gene bcl-2 exhibited decreased expression in the drug treatment groups (*p* < 0.05). Additionally, the nanogel treatment prominently induced the expression of the tumor suppressor gene p53 and p21 (*p* < 0.05). These findings are represented in Fig. 15.

**Figure 16:**
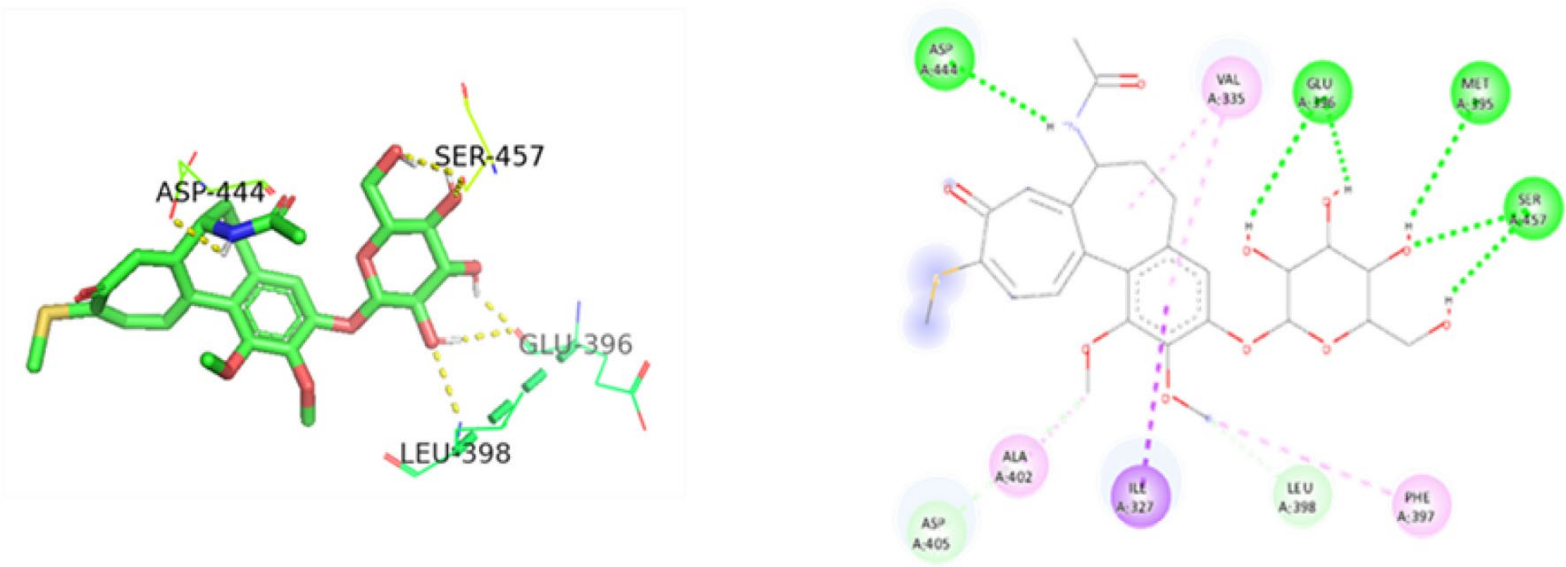
3D and 2D Ligand interaction diagram of thiocolchicoside against PAK4.

**Figure 17:**
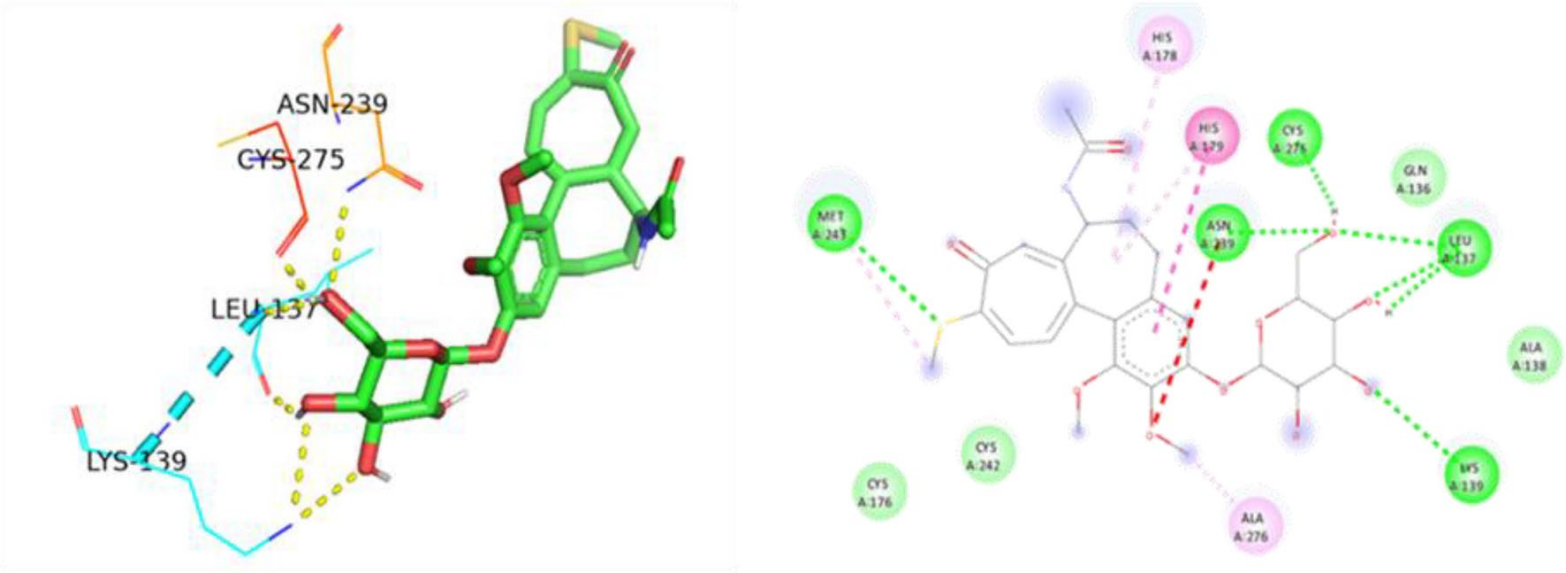
3D and 2D Ligand interaction diagram of thiocolchicoside with wildtype p53.

**Figure 18:**
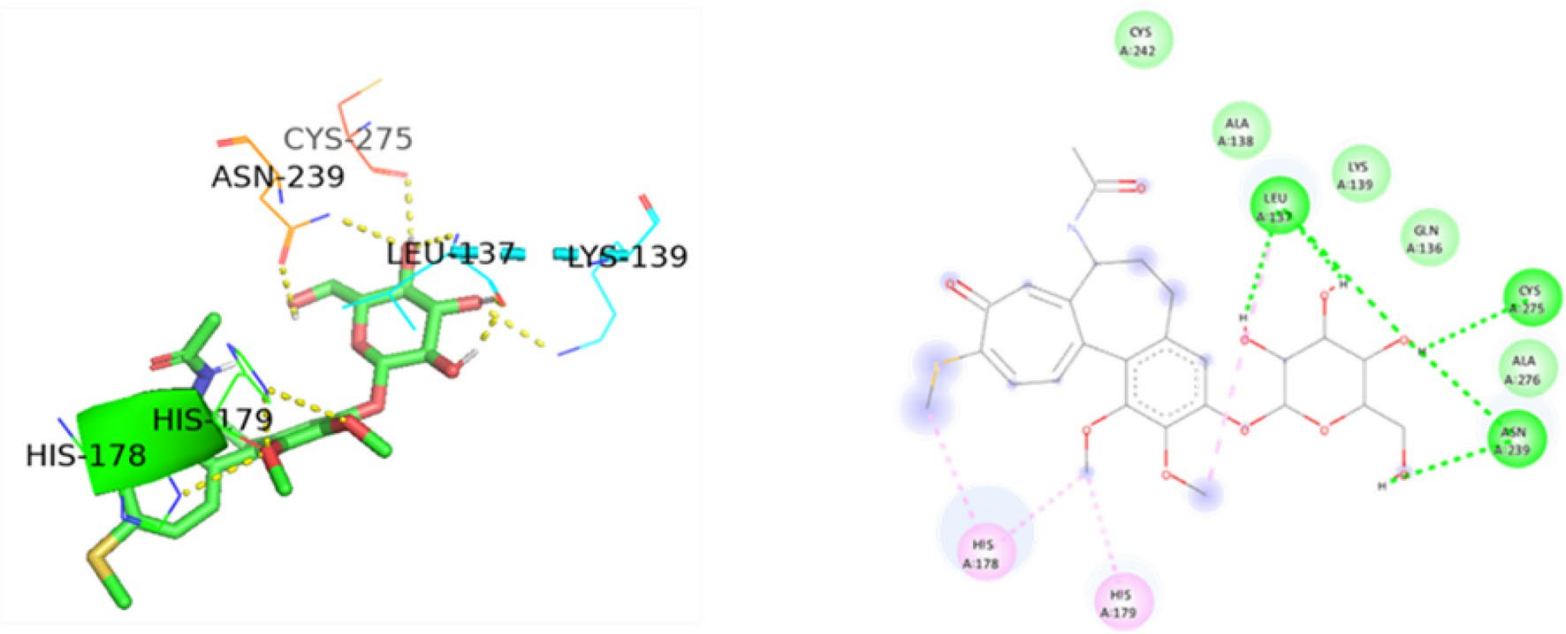
3D and 2D Ligand interaction diagram of thiocolchicoside against p53 mutant R174H.

**Figure 19:**
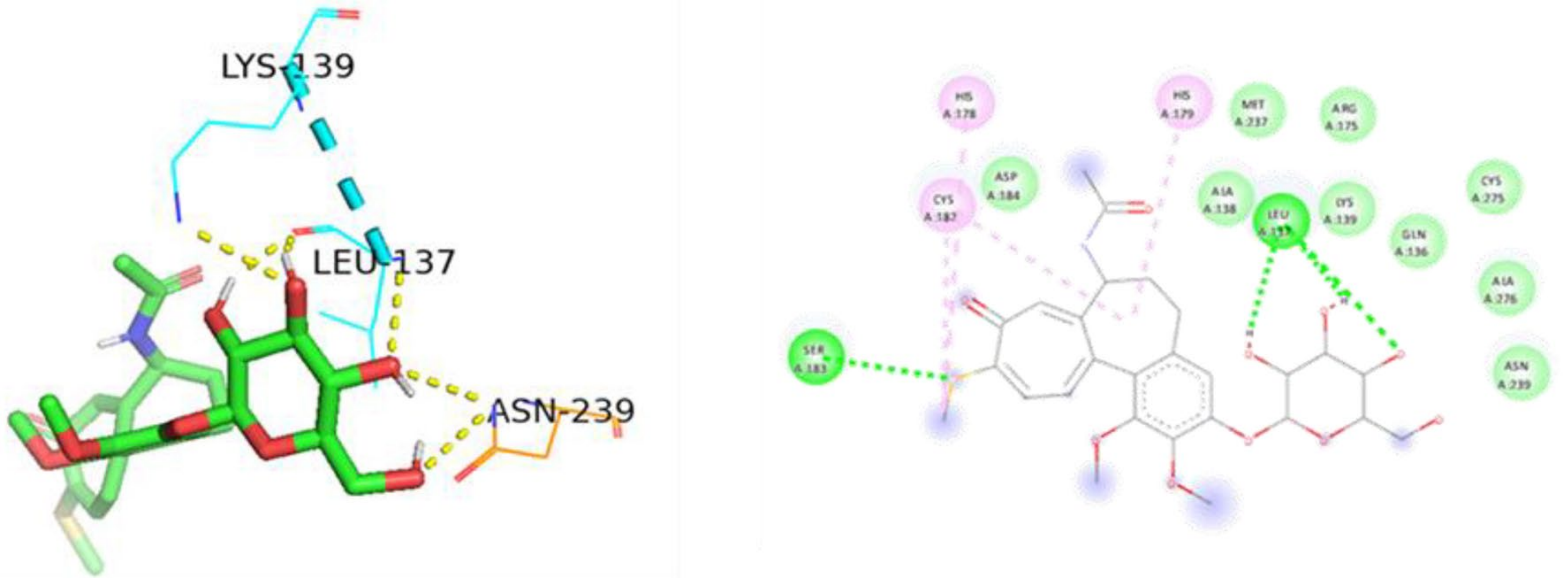
3D and 2D Ligand interaction diagram of thiocolchicoside against p53 mutant R248Q.

**Figure 20:**
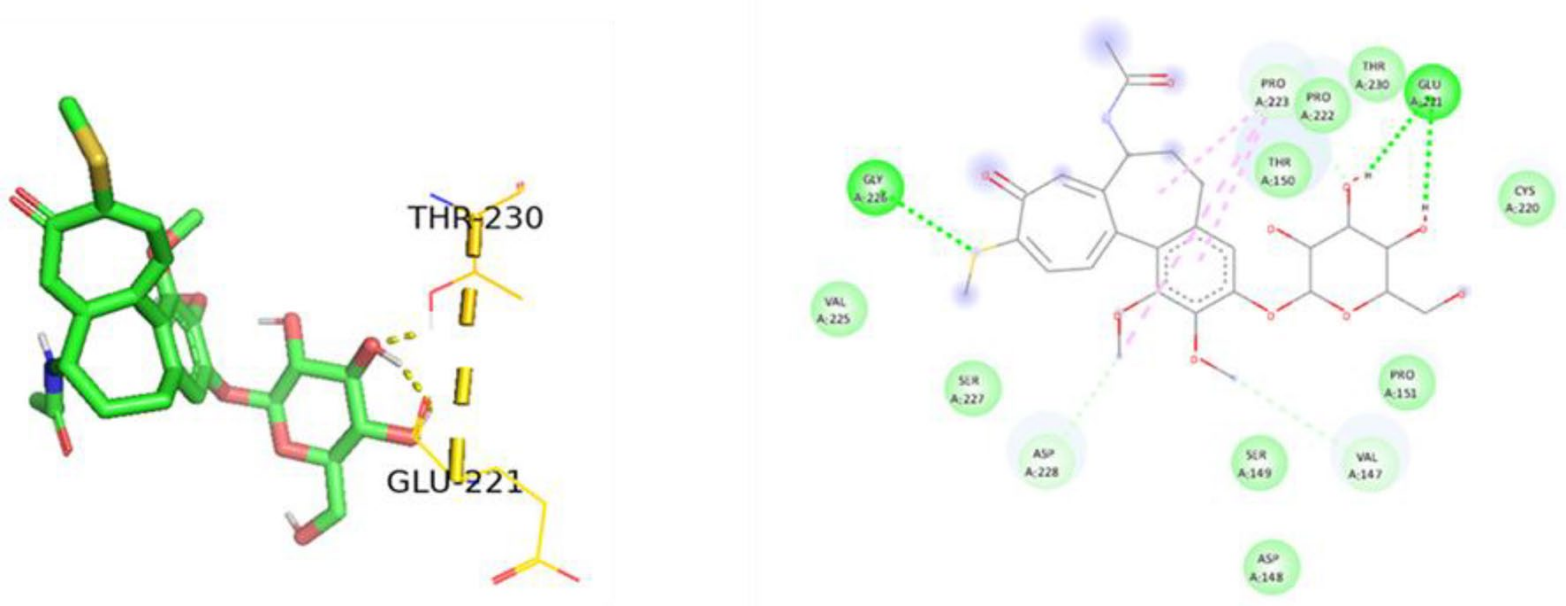
3D and 2D Ligand interaction diagram of thiocolchicoside against p53 mutant Y220C.

### The tumour-suppressing activity of thiocolchicoside against therapeutic cancer targets

Thiocolchicoside exhibited a binding affinity of − 7.28 when docked with PAK4. It established hydrogen bonds with ASP 444, GLU 396, and SER 457 and formed a carbon-hydrogen bond with LEU 398. For TP53's wildtype chain A, thiocolchicoside displayed a binding affinity of − 5.47. It engaged in hydrogen bonding interactions with ASN 239, LEU 137, CYS 275, and LYS 139 and exhibited Pi-Pi stacking with HIS 179. This binding site's proximity to the zinc-binding site suggests a potential influence on TP53's DNA binding ability, potentially enhancing its anticancer activity. Similarly to wildtype TP53, the binding of thiocolchicoside to the R248Q mutant and R175H established hydrogen bonds with LEU-137, LYS-139, and ASN-239, with slight variations in binding energy observed (Table 3). Thiocolchicoside also exhibited binding to the Y220C mutant, displaying a binding affinity of − 5.66. Within this interaction, a hydrogen bond is established with THR-230 and GLU-221. This suggests that thiocolchicoside, the active ingredient of CTL nanogel, can stabilize the protein's function or potentially restore it as a tumour suppressor (Figs. 16, 17, 18, 19, 20).

**Table 3:** Demonstrating the binding energy of gene P21 and P53.

S. no.	Target name	Complex	Binding energy	Interacting amino acids
1	P21 activated protein kinases (PAK4) – 5XVA	8	− 7.28	ASP-444, SER-457, GLU-396, LEU-398
2	p53 (wildtype)-3KMD	4	− 5.47	ASN-239, CYS-275, LEU-137, LYS-139
3	p53 (Mutant-R175H)	19	− 5.16	HIS-178, HIS-179, LEU-137, LYS-139, ASN-239, CYS-275
4	p53 (Mutant-R248Q)	18	− 5.64	LEU-137, LYS-139, ASN-239
5	p53 (Mutant-Y220C)-5O1A	16	− 5.66	THR -230, GLU-221

## Discussion

FT-IR spectroscopy results showed that the nanogel exhibited characteristic peaks at various wavenumbers, which corresponded to the functional groups present in the nanogel, including hydroxyl, aliphatic –CH3 and –CH2, carbonyl, bending vibrations of –CH2 and –CH3 groups, ester, ether, C–O, C–C, and C–H bonds. These peaks confirmed the successful synthesis of the Thiocolchicoside-Lauric acid nanogel and provided insights into the chemical composition and structure of the nanogel. The nanogel exhibited low cytotoxicity, indicating its potential for use as a safe and effective drug delivery system^32^.

Using chitosan as a mediator in preparing the nanogel is particularly noteworthy due to its unique properties and potential to enhance drug delivery capabilities. Unlike other techniques, such as NMR spectroscopy, FT-IR spectroscopy is relatively fast and inexpensive and requires minimal sample preparation. Moreover, FT-IR spectroscopy is non-destructive, making it a valuable tool for analyzing nanogels without damaging the sample^29^. The FT-IR spectra of lauric acid and its formulations, including the thiocolchicoside-lauric acid, have been characterized in previous research studies^31^. These studies have identified specific peaks corresponding to the functional groups in lauric acid and nanogel. For instance, the FT-IR spectra of lauric acid in different media were analyzed, and two bands in 1711 and 1760 cm^−1^ in the region 1650 to 1800 cm^−1^ were observed for the C=O stretching modes of dimer and monomer of lauric acid, respectively. Similarly, the FT-IR analysis of bulk lauric acid and microencapsulated lauric acid/SiO2 revealed four major peaks that were used as reference materials for the FT-IR analysis of the nanogel. Although some studies have reported FT-IR spectra of thiocolchicoside, there is still insufficient information on the spectral characteristics of this compound, especially in the context of its various formulations, such as the thiocolchicoside-lauric acid nanogel^36–39^.

Dynamic light scattering measures the hydrodynamic diameter of any nanoparticle in solution from 0.6 to 6000 nm^40^. Using DLS, we found an average particle size in the 70–110 nm range of CTL nanogel. The advantages of this technique involve the shorter duration of the study's performance and the comparatively cost-effective estimation of particle size. The disadvantages of DLS consist of the influence of nanoparticle aggregates or dust particles affecting the particle size estimation ^40^.

Aminoleslami et al.^41^ synthesized polymeric nanogels using N-vinyl caprolactam and acrylic acid, loading them with the chemotherapeutic agent Doxorubicin. Utilizing Dynamic Light Scattering (DLS), they determined against oral cancer cell lines. Zeta potential measurements provide insights into the surface charge characteristics of nanoparticles, which are pivotal in determining their stability, cellular interactions, and drug delivery capabilities. The discussion of zeta potential in this study likely involves the investigation of the electrostatic interactions between the components of the nanogel, including chitosan, thiocolchicoside, and lauric acid, as well as their influence on cellular uptake and cytotoxicity. A comprehensive analysis of zeta potential changes before and after loading thiocolchicoside and lauric acid onto the chitosan nanogel would elucidate any alterations in surface charge density, potentially affecting nanoparticle stability and interaction with cancer cells. Moreover, the discussion may explore how zeta potential variations impact the nanogel formulation's colloidal stability, which is critical for ensuring uniform distribution and sustained release of the therapeutic agents within the oral cancer microenvironment. Overall, this study discusses zeta potential likely underscores its significance in delineating the physicochemical attributes and therapeutic potential of the chitosan nanogel formulation for combating oral cancer.

The Invitro drug release studies in saliva and plasma at different concentrations from 10, 20, 40, and 80 µg/ml for a period of 12 h showed a decline in the percentage of release from CTL nanogel with an increase in the concentration of gel which was similar to a study by Swain et al. using Moxifloxacin Hydrochloride *in-situ* gel ^42^. This finding can be explained based on decreased polymer concentration and lesser viscosity, resulting in more drug release and vice-versa. Entrapment efficiency study of different concentrations of CTL nanogel at 227 nm and 276 nm also showed the stability of nanogel. The high stability of surface charges indicates the highly active nature of the nanogel interaction of CTL nanogel. During the CTL nanogel synthesis, the entrapment of drug molecules in the polymer structure created a rigid matrix structure which retarded the release of the constituent drugs from the gel, as shown in the Drug release study.

The results demonstrate that the CTL nanogel exerts potent anticancer effects against oral cancer cells. MTT assay is one of most popular and reliable viability assays for evaluating anticancer activity of synthetic and natural compounds, which depend on the conversion of the substrate to chromogenic products by the action of mitochondrial reductase in live cells, which involves the conversion of water-soluble yellow dye MTT [3-(4,5-dimethylthiazol-2-yl)-2,5-diphenyltetrazolium bromide] to an insoluble purple formazan ^43,44^. In the present study, we used four different formulations of CTL nanogel, namely a. 0.25 g LA + 0.15 g TC, b. 0.5 g LA + 0.1 g TC, c. 1 g LA + 0.05 g TC, d. 0.5 g LA + 0.05 g TC were studied from 2.5 to 60 μl/ml concentration for MTT assay. There was a dose-dependent inhibition in the cell viability of KB-1 cells after 24 h for all the four formulations of nanogel, in which CTL naogel formulation d) 0.5 g LA + 0.05 g TC showed the maximum cytotoxicity against oral cancer cells. The nanogel inhibited cell viability and induced apoptosis dose-dependently with a 50% inhibitory concentration of 10.29 µl/ml. IC-50 at 10.29 µl/ml shows the effectiveness of CTL nanogel against oral cancer cells even at lesser concentrations. Sheela DL et al. ^15^ studied the viability of human hepatocellular carcinoma, colon cancer cells and murine macrophages using MTT assay. They found dose-dependent cytotoxicity towards all three cell lines by LA from 0 to 80 mg/mL after 48 h. The IC50 values of LA were found to be 46.0 + 2.08, 39.0 + 1.29, and 36.0 + 1.87 mg/mL, respectively. In another study by Verma et al.^20^, the cytotoxicity of Virgin, Processed, and fractionated coconut oil in different concentrations was investigated for 72 h using MTT and reported a prominent growth inhibition against the KB cell line by 20% of processed coconut oil and 5% of fractioned coconut oil. The antiproliferative activity of LA was further emphasized by Lappano et al.^45^ from the study against Ishikawa endometrial cancer cells and SkBr3 breast cells. The results showed significant growth inhibition of the tumour cells without affecting the normal breast epithelial cells (MCF-10A). Antitumour activity of thiocolchicoside against squamous cell carcinoma, colon, kidney, leukemia, myeloma and breast cancer cells was reported by Reuter et al.^14^. Kiran et al. reported the cytotoxic effects of fungal-derived silver nanoparticles in various concentrations from 1.75 to 50 μl/ml on oral cancer cell lines (SCC-9) using an MTT assay. They found a 50% inhibitory concentration at 12 μl/ml^46^. Wimardhani et al. compared the effects of cisplatin and low molecular weight chitosan on Ca9-22 oral cancer cells using MTT assay and found a decline in the viability of Ca9-22 cells with IC50 800 ± 131.45 μg/mL (LMWC) and 8 ± 0.029 μg/mL (cisplatin) ^47^.

The IC-50 concentration of CTL Nanogel was fixed, and the quantification of apoptosis induction was performed using flow cytometry. While there was an observable rise in apoptotic cells in the presence of CTL Nanogel, the percentage of apoptotic cells was notably higher in CTL Nanogel-treated (Early and late apoptotic cells: 38.33%, Fig. 5) KB-1 cells compared to untreated KB-1 cells. The observed alterations in gene expression indicate that the nanogel triggers the intrinsic pathway of mitochondrial apoptosis, favouring proapoptotic molecules while downregulating the anti-apoptotic gene, bcl-2. Moreover, the induction of p53 suggests that the nanogel may promote tumour suppressor mechanisms in oral cancer cells. These findings align with the visual representation of the data in Figs. 9, 10, 11, 12, 13 and 14, which illustrate the significant changes in gene expression and cell cycle analysis after nanogel treatment.

Molecular docking experiments assessed the interaction between thiocolchicoside and PAK4 and TP53, as well as its mutants (Y220C, R175H, and R248Q). The binding affinities and the amino acids involved in these interactions were examined. P21-activated kinases (PAKs) are a group of relatively conserved serine/threonine proteins classified into two categories: PAK 1–3 (Group I) and PAK 4–6 (Group II). PAKs serve as downstream signalling effectors for Rho-family GTPases, playing pivotal roles in various fundamental cellular processes. These processes encompass cytoskeletal rearrangement, focal adhesion, cell motility, morphological alterations, and the progression of the cell cycle^48^.

In particular, PAK4 has garnered attention for its tendency to be overexpressed in numerous human cancer cell lines and tumors. Additionally, it plays a crucial role in regulating cell-cycle progression by influencing the cell-cycle regulatory protein CDKN1A levels and phosphorylating RAN^49,50^. Within TP53, two common mutations disrupt DNA binding and impact protein structure. Among these mutation types, six hotspots have been identified: Arg175, Gly245, Arg248, Arg249, Arg273, and Arg282. For our study, we specifically selected the most prevalent hotspot mutations, R175H and R248Q^51^. The Y220C mutant, known for its oncogenic properties, is an excellent model for exploring the development of small-molecule stabilizers. This mutation, involving the substitution of Tyr220 with Cyst, forms a confined hydrophobic pocket on the surface of the p53 DNA-binding domain (DBD)^52^. This alteration reduces the thermal stability of p53 by approximately 4 kcal/mol.

Ameena et al.^53^ reported the excellent antioxidant and anti-inflammatory properties of Chitosan thiocolchicoside lauric acid nanogel in oxidative stress-related disorders and inflammatory conditions in an in vitro analysis. Overall, the present study provides compelling evidence of the potential of the Chitosan thiocolchicoside lauric acid Nanogel as a promising therapeutic candidate for oral cancer treatment. The nanogel's ability to induce apoptosis and disrupt vital molecular pathways in cancer progression makes it a potential non-toxic chemotherapy option with added antibacterial properties. Further research and in vivo studies are warranted to validate its efficacy and safety for clinical translation in cancer nanomedicine.

## Conclusion

In this research, a nanogel containing thiocolchicoside and lauric acid was prepared using chitosan as a mediator and was successfully synthesized and characterized using FT-IR spectroscopy, and the nanogel demonstrated promising anticancer properties against oral cancer cells. Additionally, the chitosan-based thiocolchicoside-lauric acid nanogel induced oxidative stress in the cancer cells, as indicated by increased levels of ROS. Based on the results, we concluded that this chitosan-based nanogel, combined with thiocolchicoside and lauric acid, has the potential as a cytotoxic agent against oral cancer cells. The presence of a nanoparticle coating with various functional groups presents opportunities for further modifications to enhance its biological activities, including antimicrobial and anticancer effects, while also improving biocompatibility. The Molecular docking studies and the protein–ligand binding properties of thiocolchicoside, a key component of nanogel, displayed anticancer activities. The low binding energy of thiocolchicoside predicts the use of this drug for treating cancer. Given the promising properties of these nanoparticles, we recommend conducting further in vivo studies to assess their potential biomedical applications for future use.

## Data Availability

The datasets used and/or analyzed during the current study are available from the corresponding author on reasonable request.
